# Correlation-driven organic 3D topological insulator with relativistic fermions

**DOI:** 10.1038/s41467-023-37293-3

**Published:** 2023-04-20

**Authors:** Tetsuya Nomoto, Shusaku Imajo, Hiroki Akutsu, Yasuhiro Nakazawa, Yoshimitsu Kohama

**Affiliations:** 1 The Institute for Solid State Physics, the University of Tokyo, Kashiwa, Chiba 277-8581 Japan; 2 Graduate School of Science, Osaka University, Toyonaka, Osaka 560-0043 Japan

**Keywords:** Topological insulators, Electronic properties and materials

## Abstract

Exploring new topological phenomena and functionalities induced by strong electron correlation has been a central issue in modern condensed-matter physics. One example is a topological insulator (TI) state and its functionality driven by the Coulomb repulsion rather than a spin-orbit coupling. Here, we report a ‘correlation-driven’ TI state realized in an organic zero-gap system α-(BETS)_2_I_3_. The topological surface state and chiral anomaly are observed in temperature and field dependences of resistance, indicating a three-dimensional TI state at low temperatures. Moreover, we observe a topological phase switching between the TI state and non-equilibrium Dirac semimetal state by a dc current, which is a unique functionality of a correlation-driven TI state. Our findings demonstrate that correlation-driven TIs are promising candidates not only for practical electronic devices but also as a field for discovering new topological phenomena and phases.

## Abstract (web summary)

Topological properties can theoretically be generated by electron correlation rather than spin-orbit coupling. Here, the authors report a correlation-driven topological insulator state in the organic material α-(BETS)_2_I_3_, and its current-driven switching to a Dirac semimetal state.

## Introduction

**Fig. 1:**
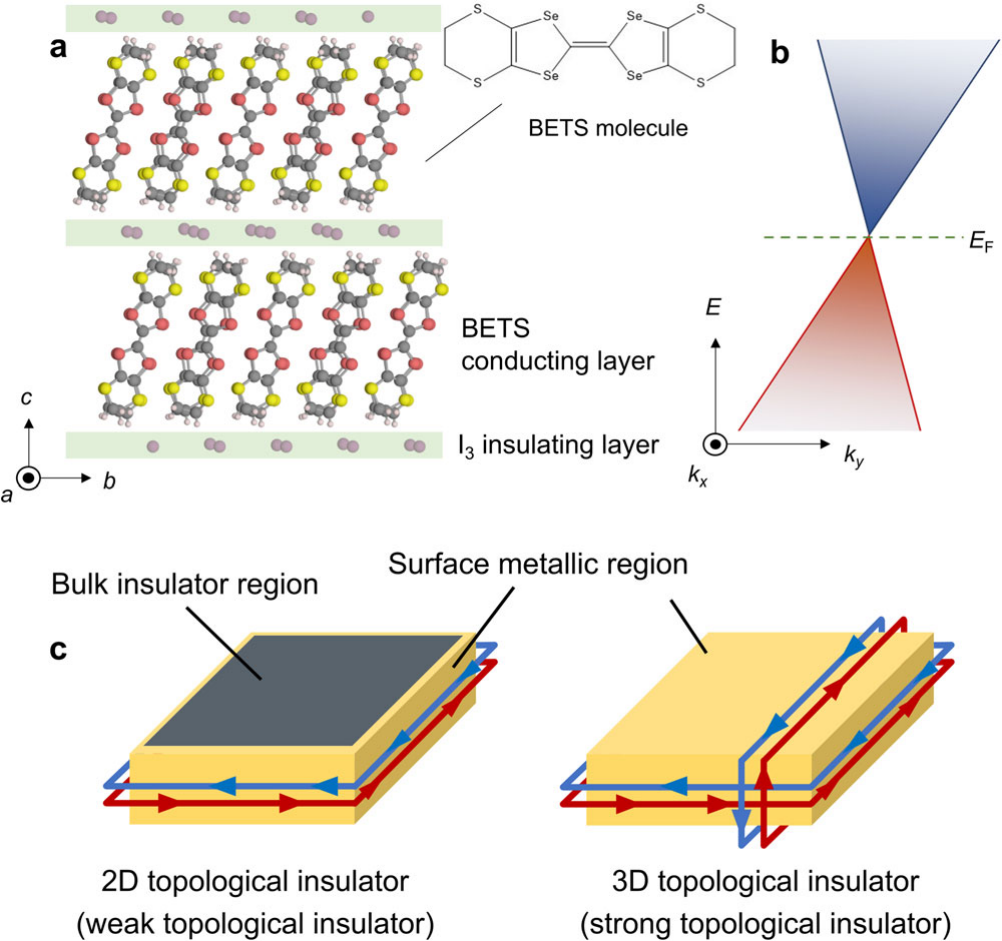
Crystal structure and predicted electronic states of α-(BETS)_2_I_3_. **a** Side view of the crystal structure of α-(BETS)_2_I_3_. BETS molecules forming 2D-conducting layers (*ab*-plane) are separated by non-magnetic insulating layers of I_3_. **b** Schematic of the band structure of α-(BETS)_2_I_3_. The valence and conduction bands are touching at the Dirac point and Fermi energy *E*_F_ is at the Dirac point. The Dirac cone is slightly tilted because of the low symmetry of the crystal^9^. **c** Schematic of 2D- and 3D-TI states in α-(BETS)_2_I_3_. In the former, the metallic surface appears on the edge of the insulating *ab*-plane. In the latter, the metallic surface appears on all the crystal faces.

Topological insulator (TI) is a new class of materials that possess both bulk insulating and exotic surface/edge states^1^. Realizing a TI state usually requires a strong spin–orbit coupling (SOC) of heavy elements, which induces a band inversion and an insulating bulk gap^2^. Thus, it is generally challenging to observe a TI state and its unique physical features in materials composed of light atoms, such as carbon-based systems, because of their weak SOC. Although several materials, such as two-dimensional organometallic systems^3,4^, have been theoretically proposed as candidates for TIs consisting of light atoms, no real system has been confirmed experimentally at present. Recent theoretical investigations propose another approach to realize a TI state using a strong electron correlation where the Coulomb interaction opens the bulk gap^5^. Such a correlation-driven TI is significant in science because it broadens the range of materials that can form TI states and has the potential to add new functions unique to correlated systems. Strongly correlated organic conductors with a topological band structure are promising candidates for this type of TIs. Here, we report a three-dimensional (3D) TI state realized in a quasi-two-dimensional (quasi-2D) organic conductor α-(BETS)_2_I_3_ (Fig. 1a) and its anomalous transport properties, where BETS denotes bis(ethylenedithio)tetraselenafulvalene^6^.

α-(BETS)_2_I_3_ consists of the conducting BETS layers and the non-magnetic insulating I_3_ layers. The BETS layers accommodate one hole for every two BETS molecules, resulting in a 1/4-filled hole band system. First-principles calculations suggest that the band structure of this compound has a Dirac-cone type linear dispersion with a narrow bandgap of *Δ* ~ 2 meV owing to SOC of Se atoms, and the Fermi energy crosses near the Dirac point (Fig. 1b)^7,8^. α-(BETS)_2_I_3_ exhibits a semimetallic behavior from room temperature and undergoes a metal–insulator (MI) transition at 50 K^9^. The prior X-ray spectroscopy and nuclear magnetic resonance (NMR) studies suggest no long-range order of charge and spin degrees of freedom, where the space inversion symmetry is not broken even below 50 K^6,10–12^. This behavior is different from the analog compound, α-(BEDT-TTF)_2_I_3_ which shows a long-range charge order (CO) at ambient pressure^13^. A recent theory suggests that the Coulomb interaction enhances the bandgap of α-(BETS)_2_I_3_ below 50 K and leads to the MI transition around 50 K, resulting in a more robust TI state with keeping the inverted band structure^14^. The concept of the correlation-driven TI state can also explain the properties of α-(BETS)_2_I_3_, such as the anomalous magnetic susceptibility^12^ and the pressure dependence of the Berry curvature^15^. Thus, the correlation-driven TI state is a candidate for the low-temperature insulating state of α-(BETS)_2_I_3_. According to the theories proposed to date, α-(BETS)_2_I_3_ is expected to behave as a stacked 2D-TI (weak 3D-TI) with a gapless state on the edge of the conducting plane (*ab*-plane) (Fig. 1c). However, experimental evidence of the 2D-TI state such as a quantum spin Hall effect has not been observed.

## Results

**Fig. 2:**
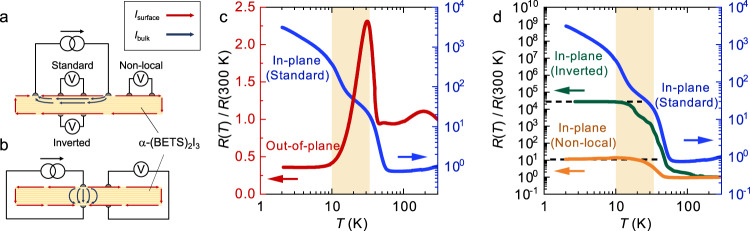
Measurement of the temperature dependence of resistance in α-(BETS)_2_I_3_. **a** Standard and inverted configuration for the four-terminal in-plane resistance measurement. Excitation currents were applied from the two terminals on the top surface of the crystal, and the voltage drop between the two terminals on the top surface (standard configuration) and a back surface (inverted configuration) was measured. The red and blue arrows represent the current flows via the surface and the bulk, respectively. **b** Setup for the out-of-plane resistance measurement. **c** Temperature dependence of the standard in-plane resistance (blue curve) for Sample #1 and the out-of-plane resistance (red curve) for Sample #2. Both data are normalized by the resistance value at 300 K. The out-of-plane resistance exhibits a drop between 35 and 10 K (orange region), indicating the appearance of the metallic surface conduction. **d** Temperature dependence of the in-plane resistance of α-(BETS)_2_I_3_ measured by the standard configuration for Sample #1 (blue curve), the inverted configuration for Sample #3 (green curve), and the non-local configuration for Sample #4 (orange curve), respectively. The resistance measured by the inverted and non-local configurations shows a flattened resistance at low temperatures because of the large contribution of the surface metallic state.

We carefully performed electrical resistance measurements by the setups shown in Fig. 2a, b to verify the accurate low-temperature electronic state of α-(BETS)_2_I. Figure 2c shows the temperature dependence of the in-plane (*I* // *ab*-plane) and out-of-plane (*I* // *c*-axis) resistances of α-(BETS)_2_I_3_. The in-plane resistance (Sample #1) slightly decreases with decreasing temperature and an exponential increase is observed at the MI transition temperature of 50 K. Furthermore, from 50 to 35 K, *R*(*T*) can be fitted well using the Arrhenius equation *R*(*T*) = *R*_0_ × exp(*Δ*/*k*_B_*T*), with an estimated gap size of *Δ* ~ 30 meV, which agrees with previous reports^9^. Since the gapless state exists only on the small area of the sample edges and most parts of the crystal behave as a band insulator in the stacked 2D-TI state, it is natural that the resistance increases following the Arrhenius law. However, the slope of the in-plane resistance becomes smaller and saturates at lower temperatures, with a step-like anomaly observed between 35 and 10 K (orange region). Such saturation of resistance is often observed in a 3D-TI with a surface conduction^16,17^, which is a different character from that expected for 2D-TIs. The out-of-plane resistance (Sample #2) is even more anomalous; an abrupt drop between 10 and 35 K and a temperature-independent behavior below 10 K are observed, while a sharp increase is observed at 50 K. Previous bulk measurements, such as the magnetic susceptibility, NMR, and lattice constant measurements, have not detected any anomalies and phase transitions near 35 K^8,10–12^. Therefore, the anomaly in the in- and out-of-plane resistances suggests the emergence of surface conduction around 35 K. This indicates that a dimensional crossover from 2D to 3D occurs at this temperature and the actual low-temperature electronic state of α-(BETS)_2_I_3_ is a 3D-TI state (Fig. 1c) despite theoretical predictions^8,14^. The dimensional crossover in TIs has been theoretically predicted, for example, in cold-atomic gases in optical lattices^18^ or quasi-2D layered systems^19^, but this is the first experimental observation in solids.

To corroborate the presence of the metallic surface state associated with the 2D–3D crossover, the in-plane resistances were measured using three different configurations; i.e., the standard four-terminal, inverted, and non-local configurations (Fig. 2a). In the inverted configuration, where the current and voltage terminals were separately attached to the top and bottom surfaces of the crystal, the current flow near the bottom surface is mostly attributed to the surface conduction. In the non-local configuration, where both the current and voltage terminals were attached on the top surface, the observed resistance is expected to exclude the bulk contribution and to be more sensitive to the surface state than the inverted configuration^16,20^. Figure 2d shows the comparison of the temperature dependences of the in-plane resistance measured by the standard configuration for Sample #1 (blue), the inverted configuration for Sample #3 (green), and the non-local configuration for Sample #4 (orange). For the inverted and non-local configurations, flattened resistance curves are observed below 10 K, which is characteristic for 3D-TIs where the conductivity is dominated by the surface contribution^16,17^, but not for 2D-TIs without surface conduction. In addition, the observed resistance in the non-local configuration monotonically increases around 35 K and becomes flattened below 10 K, whereas the resistance curves in the standard and inverted configurations show step-like anomalies in this temperature range. These results indicate the emergence of the surface contribution and are consistent with our interpretation that the 2D–3D crossover occurs between 35 and 10 K. The saturating tendency of resistance observed in the standard configuration below 10 K can be attributed to the combination of the exponential increase of the bulk state resistance and the constant value of the surface resistance. More detailed comparisons between the in-plane and out-of-plane resistance and discussions on the sample dependence are provided in Sections 1 and 2 of Supplementary Information.

Next, we performed magnetoresistance (MR) measurements up to high magnetic fields to reveal the topological character of α-(BETS)_2_I_3_. If α-(BETS)_2_I_3_ is a 3D-TI with a narrow band gap at low temperatures, the thermally excited carriers are expected to exhibit the unique properties of relativistic 3D Weyl fermions due to the Dirac-cone type band dispersion of this compound^21,22^. One of the characteristics of such relativistic fermions is the quantum transport phenomenon caused by the degree of freedom of chirality. In the quantum field theory, massless fermions are divided into left- (clockwise) or right-handed (anti-clockwise) particles. In the absence of any external gauge field, the massless fermions with opposite chirality do not mix with each other, and the balance of chirality is conserved. However, on the application of an electric current (*I*) and magnetic field (*B*) in parallel (*I* // *B*), the symmetry of the chiral fermions is broken. In general, this results in the Adler–Bell–Jackiw chiral anomaly or chiral magnetic effect (CME)^21–24^, where a large negative MR is observed. This CME-induced negative MR is known as critical transport evidence for the presence of relativistic fermions and is observed in inorganic topological semimetals such as Na_3_Bi^25^ and Cd_3_As_2_^26^, and strong 3D-TIs with a linear band dispersion such as ZrTe_5_^27–29^.

**Fig. 3:**
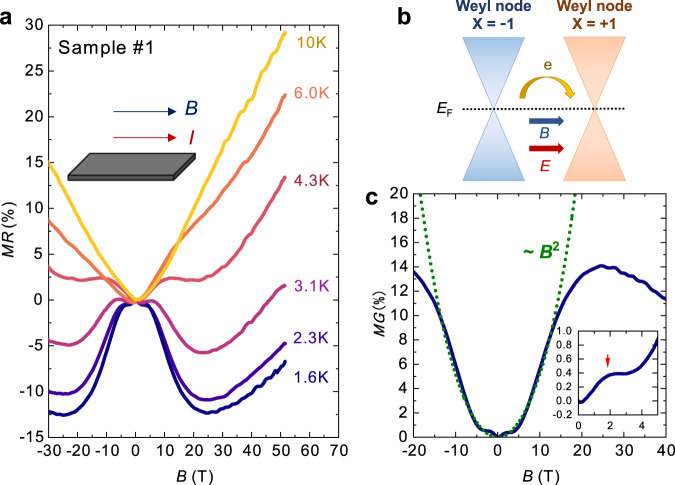
Negative magnetoresistance (MR) in α-(BETS)_2_I_3_. **a** Longitudinal MR of α-(BETS)_2_I_3_ (*I* // *B*). The magnetic-field dependence of MR was measured at several temperatures. The quadratic negative magnetoresistance can be observed below 4 K. **b** Schematic of the charge flow induced by CME. In a magnetic field, the positive magnetoconductance (MG) due to the chiral anomaly flows between two Weyl nodes with different chirality *x* = ±1. **c** MG at 1.6 K. The green dashed line denotes the fitting curve MG = *C*_a_*B*^2^ with *C*_a_ = 4.92 × 10^−4^. Below 15 T, MG follows *B*^2^ dependence, indicating the CME-induced MR. Inset: Expanded figure of MG at 1.6 K. The hump-like positive MG can be observed below 5 T, implying weak localization.

Figure 3a shows the normalized in-plane MR of α-(BETS)_2_I_3_ (Sample #1) in the configuration of *I* // *B*, which results in a negligible effect of the Lorentz force on MR. In the high-temperature region (*T* > 10 K), a positive MR monotonically increases with *B*. However, a dip-like anomaly is observed below 25 T with a further decrease in the temperature (*T* < 10 K). Finally, the dip structure converts into a negative MR below 4 K. At the lowest temperature of the measurement (*T* = 1.6 K), the negative MR reaches −14% at *B* = 25 T. Although a large negative MR is often observed in magnetic molecular compounds owing to electron-spin scattering^30^, α-(BETS)_2_I_3_ is non-magnetic. Thus, the negative MR in α-(BETS)_2_I_3_ can be attributed to CME. In the case of Dirac band systems, CME induces a charge flow in the direction of *I* (and *B*) to compensate for the chirality imbalance between the two Weyl nodes with distinct chirality *x* = +1 and *x* = −1 (Fig. 3b). In theories of CME^31,32^, the positive magnetoconductance (MG) in weak magnetic fields is expressed as MG ~ MR^−1^ ~ *C*_a_*B*^2^, where *C*_a_ is a fitting parameter. Figure 3c shows the magnetic-field dependence of MG and the results of the quadratic fitting. The consistency of the fitting in a wide magnetic field range (*B* < 10 T) suggests that the positive MG (negative MR) in α-(BETS)_2_I_3_ is induced by CME. Above ~25 T, MG exhibits a downturn behavior. Because the magnetic field was applied parallel to the 2D conduction layers, the effects of the Landau level splitting and quantum limit are considered to be small^31–33^. Thus, the downturn observed in the high-field region is probably caused by the transverse MR or Hall component owing to the slight misalignment of *B*. Moreover, an additional positive MG of less than 1% in weak magnetic fields (*B* < 5 T) was observed, as shown in the inset of Fig. 3c. Owing to its extremely small value and tendency to appear only at low temperatures and weak magnetic fields, it is considered to be a contribution of the weak localization effect due to the impurities^12^. These results demonstrate that the conduction electrons in α-(BETS)_2_I_3_ behave as 3D Weyl fermions. The original band structure in this compound has a Dirac-cone-type linear dispersion, where the small band gap of *Δ* ~ 30 meV is induced by electron correlations. Therefore, the thermally excited electrons can hold a character of the chiral fermions^29^. A detailed discussion on the negative MR is in Section 3 of Supplementary Information.

**Fig. 4:**
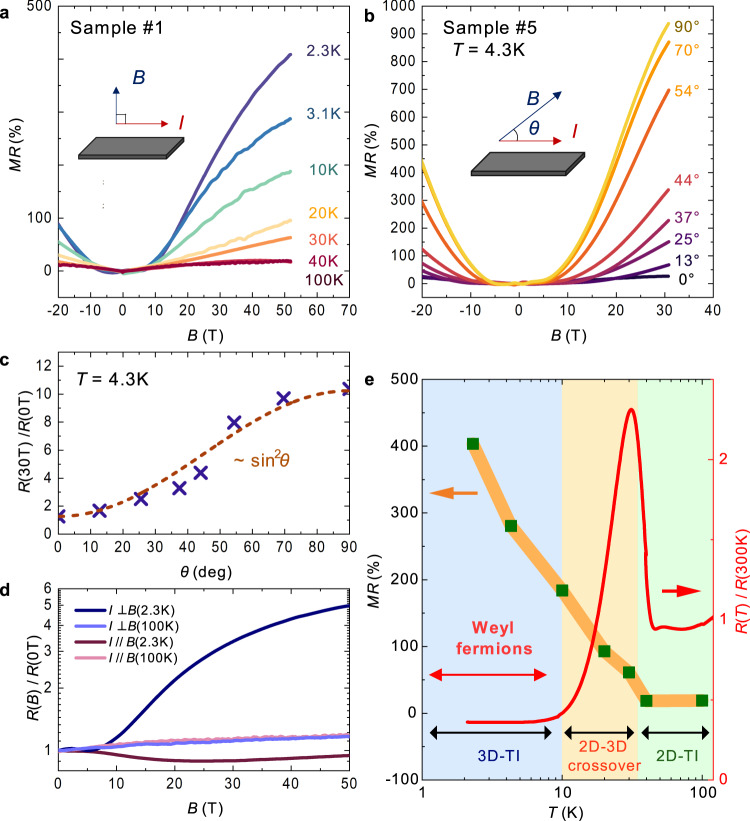
Positive and non-saturating MR in α-(BETS)_2_I_3_. **a** Transverse MR of α-(BETS)_2_I_3_ (*I*⊥*B*). The magnetic-field dependence of MR of Sample #1 was measured at several temperatures. **a** Large positive and non-saturating MR can be observed. **b** The magnetic field dependence of MR of Sample #5 at 4.3 K measured at several angles *θ*. The angle *θ* is defined by the angle between *I* and *B*. **c** Angle dependence of MR of Sample #4 at 30 T. The orange dashed line is the fitting line of sin^2^*θ*. **d** Comparison of MRs of Sample #1 of *I* ⊥ *B* and *I* // *B* at 2.3 and 100 K, respectively. The strong anisotropy of MR can be observed only at low temperatures. **e** Temperature dependence of the magnitude of the transverse MR of Sample #1 and the out-of-plane resistance of Sample #2. Below 35 K, the positive MR begins to increase, which agrees with the appearance of the surface conduction on the *ab*-plane.

Another characteristic magnetotransport property of Dirac systems is a large positive MR effect in the *I*⊥*B* configuration^34–37^. Figure 4a shows the magnetic field dependence of MR of Sample #1 when *B* is perpendicular to the *ab*-plane. In contrast to the *I* // *B* configuration, a large and non-saturating positive MR is observed. The positive MR increases with decreasing temperature and exceeds 400% at 2.3 K and 50 T. In addition, it shows *B*^2^-dependence at lower magnetic fields (*B* < 20 T) and a sublinear dependence at higher magnetic fields (*B* > 20 T). Such a non-saturating MR is characteristic of compensated metals, such as Dirac semimetals, wherein holes and electrons compensate each other, thereby resulting in a positive MR effect by the Lorentz force that continues to appear in high magnetic fields. Indeed, the positive MR can be qualitatively reproduced using the two-carrier model as well as other topological semimetals^37,38^ (see Supplementary Information). Figure 4b shows the MR of Sample #5 as a function of the elevation angle *θ* between *B* and *E*, measured at 4.3 K. Herein, the negative longitudinal MR by CME is negligibly small compared to the positive transverse MR at this temperature. When *θ* = 0°, MR exhibits the smallest value owing to the Lorentz force-free configuration. Thereafter, with an increase in *θ*, MR increases monotonically, reaching the maximum value at *θ* = 90°. Such a field-direction-sensitive MR is a specific feature universally observed in Dirac/Weyl fermion systems^26,27,34–36^. The relationship between the magnitude and angle of MR (Fig. 4c) can be fitted well with a curve proportional to sin2*θ* (the orange dashed line); thus, the origin of the transverse MR in α-(BETS)_2_I_3_ is the classical Lorentz force. Comparing the MR magnitudes at 2.3 and 100 K for the *I* // *B* and *I*⊥*B* configurations (Fig. 4d) reveals that no anisotropy of MR can be observed at 100 K (the metallic state). Therefore, the anisotropy of MR originates from the low-temperature electronic state rather than the anisotropy of the crystal structure. Since 3D chiral fermions are not produced by a thermal disturbance in the high-temperature 2D-TI state, it is natural that the negative MR cannot be observed at high temperatures. This is in contrast to that of inorganic 3D topological semimetals, where the CME-induced negative MR can be observed at high temperatures. The temperature dependences of the magnitude of the transverse MR of α-(BETS)_2_I_3_ and the out-of-plane resistance are plotted in Fig. 4e. The temperatures where the surface conduction emerges agree with the temperature where the transverse MR begins to increase, suggesting the appearance of a topological surface state with Dirac-type band dispersion near 35 K. In addition, the CME-induced negative MR is observed below 10 K (Fig. 3a), where the out-of-plane resistance exhibits a temperature-independent behavior. This indicates that the middle-temperature region between 35 and 10 K is a crossover regime from 2D- to 3D-TI, where the 3D Weyl fermions are absent or very few in number. Moreover, no Shubnikov–de Haas oscillations are detected during measurements. It is considered that the absence of quantum oscillations is owing to the coincidence of the Fermi energy with a Dirac point. Indeed, quantum oscillations have been observed on carrier-doped samples in relatively weak magnetic fields below 8 T^15^.

The anomalous MR properties indicate that α-(BETS)_2_I_3_ is a correlation-driven 3D-TI. A significant difference from conventional TIs is that the bulk insulating state is sustained by the electron correlation. It is known that the conventional insulating states derived from the electron correlation, such as Mott insulators and charge-ordered (CO) insulators, can be electrically controlled using a dc current^39,40^. Similarly, a correlation-driven TI state is expected to be controlled by a dc current and be changed into another topological state. Such electrical controllability of topological matters would further broaden practical applications for electronic devices. Here, we challenge to control the correlation-driven TI state of α-(BETS)_2_I_3_ by an external dc current.

**Fig. 5:**
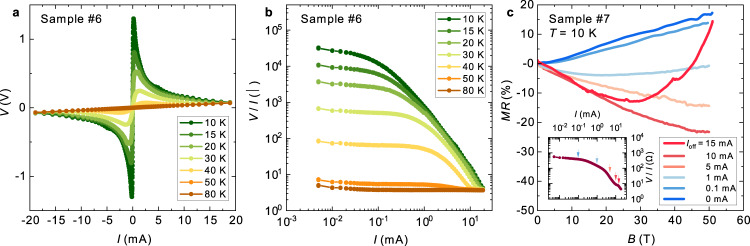
Current switching of the topological phase in α-(BETS)_2_I_3_. **a** Current–voltage (*I* –*V*) characteristic of Sample #6 measured at several temperatures using the four-terminal dc method with constant currents. A nonlinear conduction effect is observed in the wide current range. **b** Current dependence of the sample resistance (*V*/*I*). In the low current region, *V*/*I* is a constant value followed by Ohm’s law. As the applied current increases, the sample resistance decreases by several orders of magnitude. **c** Longitudinal MR of Sample #7 (*I* // *B*) at 10 K measured under the several values of the offset dc current (*I*_off_). The positive to negative change of MR indicates the topological phase switching from TI to the Dirac semimetal state. Inset: the *I*–*V/I* character of Sample #6. Nonlinear conduction is clearly observed above *I* > 0.1 mA.

Figure 5a, b show the current–voltage (*I*–*V*) characteristic and current dependence of the resistance of Sample #6 under constant current conditions, respectively. These measurements were performed under the exchange gas condition using a pulsed current for <10 ms to avoid the influence of Joule heating. We observe a giant nonlinear conduction effect at temperatures below the MI transition (Fig. 5a). The sample resistance (*V*/*I*) decreases with increasing current, and a drastic reduction of approximately four orders of magnitude in resistance is observed at 10 K (Fig. 5b). Such an anomalous *I*–*V* characteristic is reminiscent of those of a tunnel diode or correlation-driven insulators^39^. The *I*–*V* curves show a local peak at some voltages (*V*_peak_); for instance, *V*_peak_ is 0.072 V (=7.2 V/cm) at 40 K (Fig. S5a). Note that the value of *V*_peak_ almost corresponds to the threshold voltage *E*_th_ of nonlinear conduction^40,41^. Based on the Zener breakdown model^42^, which is a classical mechanism for nonlinear conduction, *E*_th_ is estimated as *E*_th_ ~ *Δ*/*ea*, where *Δ* is the gap size, *e* is the elementary charge, and *a* is a lattice parameter. Using *a* ~ 1 nm from crystallographic data^9^ and *Δ* = 30 meV, *E*_th_ is estimated to be ~0.3 MV/cm, which is clearly larger than the order of *V*_peak_. The value of *V*_peak_ exponentially increases with decreasing temperature, which is observed in the charge-density-wave (CDW) sliding and Mott gap suppression by a current; a thorough discussion is available in Supplementary Information. The *E*_th_ value for the depinning of CDW is usually 10^−1^–10^−2^ V/cm, which is clearly smaller than the estimated *E*_th_ value for this compound. Therefore, the origin of the nonlinear conduction of α-(BETS)_2_I_3_ is supposed to be the bandgap suppression in the bulk region similar to the nonlinear conduction in Mott insulators. Here, the application of a dc current may reduce the gap size of the correlation-driven TI state, leading to a different non-equilibrium electronic state.

When the bandgap on the Dirac-cone is closed by applying a large current, the bulk Dirac semimetal state likely emerges, which exhibits the anomalous magneto-transport properties caused by relativistic chiral fermions. We investigated the current dependence of MR to clarify the nature of carriers in the possible current-induced Dirac semimetal state in α-(BETS)_2_I_3_. Figure 5c displays the magnetic-field dependence of the longitudinal MR (*I* // *B*) measured at 10 K using the four-terminal ac method with a dc offset current *I*_off_. When no *I*_off_ is applied, a positive MR is observed at 10 K, which is consistent with Fig. 3a. As the current is increased, the positive MR is gradually suppressed and turns to negative MR above *I*_off_ > 1 mA (=27 A/cm^2^). The positive/negative switching in MR is a unique feature of this material that has not been observed in conventional nonlinear conduction materials^43^. With a large dc current (*I*_off_ = 15 mA), MR exhibits an upturn above ~30 T, which is comparable to the longitudinal MR with no offset current at lower temperatures (Fig. 3a). Such current dependence of MR cannot be explained by the Joule heating effect. Furthermore, the CME-induced MR is negligible because this result was obtained at 10 K. Therefore, the negative MR can be attributed to CME by the current-induced carriers in the bulk. This supports the scenario that the bandgap on the Dirac-cone is forced to be closed by applying a dc current, realizing the non-equilibrium Dirac semimetal state with chiral fermions in α-(BETS)_2_I_3_. We note that the current-induced negative MR is qualitatively reproduced in steady magnetic fields (see Section 5 in Supplementary Information for details) and is not due to the Joule heating effect. If the sample is heated up with Joule heating, the observed MR should decrease with increasing current value.

Our results suggest that a correlation-driven TI possesses electrical controllability in a different topological system. This unique feature has not been observed in conventional TIs driven by SOC. Topological materials, whose physical properties can be electrically controlled, are desirable not only as a practical electronic device but also as a system for seeking a novel topological phase and phenomenon. Consequently, our findings demonstrate that correlation-driven TIs hold the potential to be a new playground for the science of topology.

In summary, we revealed that α-(BETS)_2_I_3_ is a correlation-driven 3D-TI with relativistic fermions at low temperatures. In addition, we demonstrated the topological phase switching phenomena between the Dirac semimetal and TI by an external current. These findings provide a new direction for the development of topological materials such as TIs and Dirac/Weyl semimetals and for the exploration of their functionality concerning device applications. The origin of the 2D–3D crossover near 35 K and the detailed mechanism of bulk gap closure by applying a dc current are still open questions. The theoretical investigation of the topological characters of α-(BETS)_2_I_3_ will be presented in a future study.

## Methods

Single crystals of α-(BETS)_2_I_3_ were grown using a standard electrochemical method. The in-plane and out-of-plane resistances as shown in Fig. 2 were measured using the four-terminal method in a physical property measurement system (PPMS, Quantum Design). To perform the standard in-plane resistance and magnetoresistance (MR) measurements, four gold wires were attached to the conducting plane (*ab*-plane) using a carbon paste. In the case of the in-plane resistance measurement for Sample #3 with the inverted configuration, two gold wires were connected to the current electrodes on the top surface and the other two wires were connected to the voltage electrodes on the bottom surface. In the case of the non-local resistance measurement for Sample #4, both two gold wires for the current electrodes and the other two wires for the voltage electrodes are on the top surface. Dimensions of measured Samples #1–#7 are summarized in Table S1. Because the crystals were very thin, there is considerable ambiguity in thickness *t*. In the case of out-of-plane measurements, two gold wires were attached to the top of the *ab*-plane, whereas the other two were attached to the back.

The MR measurements in pulsed magnetic fields were performed using the standard ac four-terminal method with an excitation voltage at a frequency of 2–5 kHz. The amplitude of excitation voltage was set within the ohmic conduction range of the samples. Regarding the low-noise ac resistance measurements, the voltage signal was amplified using pre-amplifiers (Model SR560, Stanford Research Systems). Furthermore, the angle of *B* was calculated as the ratio of induced voltages of multiple pickup coils, and the temperature was measured using a calibrated Cernox thermometer and a temperature controller (Lakeshore model 335).

The current–voltage (*I*–*V*) characteristics were measured using a short pulse current of 10 ms produced by a current source (NI-9265, National Instruments) and a multimeter (Model 2000, Keithley). To avoid the self-heating effect, this measurement was performed under exchange gas conditions. We also measured the temperature dependence of resistance with five different excitation current values (1, 4, 8, 12, and 16 mA) to estimate the size of the band gap in each condition and to discuss the current-induced electronic state in detail (see Section 5 in Supplementary Information).

The current dependence of the longitudinal MR was measured using the four-terminal method with an ac excitation and a dc offset current. The dc offset current (*I*_off_) was produced by the current source NI-9265. The amplitude of the ac current was set to ~10% of the magnitude of *I*_off_ to satisfactorily avoid the change of electronic state by an ac component. Although the application of the excitation ac voltage induced a mismatch between the actual sample resistance and the estimated value from the *I*–(*V/I)* characteristic, a combination of dc and ac did not have extrinsic effects on the MR properties. Therefore, the effect of the current-induced electronic state on the magnetic response could be investigated.

To confirm that the current dependence of MR is intrinsic, we also measured it in steady magnetic fields by using PPMS (see Section 5 in Supplementary Information). In this measurement, the MR was measured by the standard dc four-terminal method by applying a short pulse of excitation current for 10 ms.

## Supplementary information

Supplementary Information

## Data Availability

Source data are provided with this paper. All other data that support the findings of this study are available from the corresponding author upon request. Source data are provided with this paper.

## Source data

Source Data
