# Supplementary material for: Correlation-driven organic 3D topological insulator with relativistic fermions
Source: Nat Commun. 2023 Apr 20;14:2130. doi: 10.1038/s41467-023-37293-3 (PMC10119126; doi:10.1038/s41467-023-37293-3)
Supplement: Supplementary file 1 — Supplementary Information [file 41467_2023_37293_MOESM1_ESM.pdf]

Supplementary Information for
'Correlation-driven Organic 3D Topological Insulator with Relativistic Fermions'

Tetsuya Nomoto[1,2,*], Shusaku Imajo[1], Hiroki Akutsu[2], Yasuhiro Nakazawa[2], Yoshimitsu Kohama[1]

[1]*The Institute for Solid State Physics, the University of Tokyo, Kashiwa, Chiba 277-8581, Japan*
[2]*Graduate School of Science, Osaka University, Toyonaka, Osaka 560-0043, Japan*

Table of Contents

§1 Comparison between in-plane and out-of-plane resistance

§2 Sample dependence and reproductivity of resistance

§3 Negative magnetoresistance of the 3D-TI state of α-$(BETS)_2I_3$

§4 Positive magnetoresistance in the two-carrier model

§5 Non-equilibrium metallic state in α-$(BETS)_2I_3$

Figure S1-S7

Table S1

References

§1 Comparison between in-plane and out-of-plane resistance

The impact of the surface conduction is expected to be different between the in-plane and out-of-plane resistance due to the anisotropy of bulk resistivity. First, the bulk resistivity of α-$(BETS)_2I_3$ is highly anisotropic because of the highly anisotropic crystal structure. At 300 K, for example, the in-plane resistivity is 4.6×10^{-2} Ω cm (Sample #1), and the out-of-plane resistivity is 1.2×10^{1} Ω cm (Sample #2); the out-of-plane resistivity is about 250 times larger than the in-plane resistivity. In the presence of topological surface states, the observed resistance becomes a combined resistance of bulk and surface resistances. Thus, the influence of anisotropy can be seen in the temperature dependences of the in-plane and out-of-plane resistivity. Since the bulk resistivity of the out-of-plane direction is much higher than that of the in-plane direction, the observed out-of-plane resistance is influenced by the appearance of surface state more seriously than the in-plane one. Therefore, it is reasonable that the drop of resistance and temperature-independent behavior were observed clearly in the out-of-plane resistance measurement. Furthermore, the increase in the in-plane resistance below 10 K can be attributed to the nonnegligible contribution of the bulk conduction to the surface one in this temperature region. Such behavior has been observed when a TI sample has a large bulk contribution [1]. We note that the in-plane resistance measured by the inverted configuration exhibit a flattened temperature dependence similar to the out-of-plane resistance at low temperatures (Fig. 2 in the main text), because the contribution of the surface conduction is dominant in this configuration. Detailed discussions about the sample dependence and reproductivity are provided in the following section.

§2 Sample dependence and reproductivity of resistance

In this section, the reproducibility of the temperature dependence of the resistance is discussed. In a normal bulk insulator or bulk metal, the resistance is inversely proportional to the sample size. Thus, the resistivity or normalised resistance by the value at room temperature does not depend on the sample size. In contrast, when the resistivity of the bulk and surface is different as in a TI, the resistance is determined by the ratio of the bulk and surface conductance. The contribution of surface conduction can be influenced by the shape and size of samples and the arrangement of the electrodes. Therefore, the resistance of TIs cannot be simply normalised by the sample size or room-temperature resistance. The sample size dependence of the resistivity is often observed in 3D-TI [2-5].

Figure S1 shows the Arrhenius plot of the in-plane resistance of nine different samples, Sample #1 and Sample I1–I8. Above 35 K, all the data can be scaled on a single curve, suggesting the absence of surface conduction. In contrast, below 35 K, the sample dependence can be clearly observed, which strongly suggests the presence of surface conduction below 35 K.

In the case of the out-of-plane resistance, the sample dependence is more complicated. Although the resistance drop at approximately 35 K can be reproduced in a different sample (Sample O1), as shown in Fig. S2a, several samples did not show a clear resistance drop, as shown in Fig. S2b (Sample O2). We consider that the discrepancy of temperature dependence of the out-of-plane resistance at low temperatures is also due to the surface state. The temperature dependence of the out-of-plane resistance, which can be affected by the surface state more seriously than that of the in-plane resistance, varies from sample to sample, resulting in the bad reproducibility. Conversely, the temperature dependence of the out-of-plane resistance in the high-temperature region, where no surface

conduction exists, is reproducible. For example, the low-temperature temperature dependence of the out-of-plane resistance of Sample #2 and Sample O2 is quite different, but above 35 K, where no metallic state exists, they agree relatively well as shown in Fig. S2b.

§3 Negative magnetoresistance in the 3D-TI state of α-$(BETS)_2I_3$

In this section, we discuss the quadratic negative magnetoresistance (MR) of α-$(BETS)_2I_3$. As mentioned in the main text, the quadratic negative MR is widely observed in bulk 3D Dirac/Weyl semimetals. Thus, it might be possible to consider that the low-temperature electronic state of this compound is a bulk Dirac semimetal state, not a 3D-TI state. However, for the reasons discussed below, we believe this compound to be a 3D-TI state at low temperatures.

The first reason is the temperature dependence of resistance. In the Dirac/Weyl semimetals reported in previous works, the resistance typically shows temperature-independent or metallic behaviour at low temperatures [6, 7]. In contrast, the resistance of α-$(BETS)_2I_3$ increases by several orders at low temperatures with nearly saturated behaviour. This behaviour is rather similar to the temperature dependence of topological Kondo insulators [1], where the resistance of the sample increases as decreasing temperature with the gap opening of an insulating bulk state.

The second reason is the MR in the out-of-plane resistance. If this compound is a bulk Dirac semimetal at low temperatures, the negative MR should be observed in the out-of-plane resistance when a magnetic field is applied parallel to the current direction (B // I). However, as shown in Fig. S3, no negative MR was observed even at 2 K with applying magnetic field of 10 T for B // I. This is obviously different from the MR taken with the

out-of-plane configuration on the analogue compound, α-(BEDT-TTF)$_2$I$_3$, which is a bulk Dirac semimetal under pressure [8]. If α-(BETS)$_2$I$_3$ is a 3D-TI where the bulk is an insulator and the currents flows along the topological surface, it is reasonable not to observe a negative MR for the out-of-plane resistance, because the current flow on the sample surface cannot hold the condition for the chiral magnetic effect (B // I). Considering these characteristics, it is reasonable to judge that α-(BETS)$_2$I$_3$ is a 3D-TI at low temperatures rather than a bulk Dirac semimetal.

§4 Positive magnetoresistance in the two-carrier model

In this section, the two-carrier model mentioned in the main text is explained. In the classical model [9], the MR ΔR / R (0 T) of the two carrier systems is described by the following equation:

$$\frac{\Delta R}{R(0T)} = \frac{r_e r_h (R_e + R_h) + \left(r_e {R_h}^2 + r_h {R_e}^2\right) B^2}{(r_e + r_h)^2 + (R_e + R_h)^2 B^2} \times \frac{r_e + r_h}{r_e r_h} - 1 \qquad (1),$$

where r_e and r_h are the resistances of electrons and holes, respectively, and R_e and R_h are the Hall coefficients of the electron and hole carriers, respectively. Because the two carriers mutually depress the Hall electric field formed by the polarisation owing to the Lorentz force, MR becomes large and does not saturate until high magnetic fields. This model can explain the large MR value of materials with a semimetallic band such as graphene, Bi, and some topological semimetals [10-13].

Figure S4 shows MR when B is perpendicular to I (blue series) and the fitting curve using Eq. 1. In this analysis, it was assumed that $r_n(T) = r_h(T)$ (= $r_0(T)$). The equation can reproduce MR quantitatively, indicating that the anomalously large MR of α-(BETS)$_2$I$_3$

is induced by the Dirac-cone-type semimetallic band structure. However, the minor mismatch of the fitting below 20 T is probably because of the negative MR caused by the longitudinal component.

§5 Non-equilibrium metallic state in α-$(BETS)_2I_3$

In this section, we supplement the non-linear conduction and current-induced metallic state in α-$(BETS)_2I_3$. As mentioned in the main text, the current–voltage (I–V) curves exhibit peak structures at V_{peak} (Fig. S5a). The relationship between V_{peak} and T is shown in Fig. S5b. The V_{peak} exponentially increases with decreasing temperature, and thus it can be fitted well using $V_{peak} = V_0 \exp(-T/T_0)$, where V_0 and T_0 are the fitting parameters. Using the characteristic values V_0 = 3.18 V (= 3.18 kV/cm) and T_0 = 11.1 K, we succeeded in fitting our results. This relation is often observed in materials that exhibit nonlinear conduction induced by the electron delocalization, such as charge-density-wave and Mott insulators. The exponential increase of V_{peak} indicates that the nonlinear conduction of α-$(BETS)_2I_3$ cannot be explained by the classical models where the threshold voltage weakly depends on temperature.

In the main text, we speculated that the suppression of the bandgap on the Dirac band is the origin of the giant nonlinear conduction. Thus, we measured the excitation current dependence of the in-plane resistance using the four-terminal method to verify this speculation. Figure S6a is the temperature dependence of the in-plane resistance of Sample S1 measured by applying several external current values, and Fig. S6b is the log-log plot of Fig.S6a. For small excitation currents, the temperature dependence of the resistance reproduces the result of Sample #1 shown in Fig. 2c. As the excitation current is increased, the increase of resistance below the metal-insulator transition becomes

weaker. The transition temperature, which is determined by the onset temperature of the resistance increase as shown as the black triangles in Fig. S6b, is shifted to lower temperatures with increasing current. Such a shift is widely observed in materials showing non-linear conduction [14, 15] and is consistent with the theoretical prediction of the current effect on correlation-driven insulators [16]. Finally, a temperature-independent resistance is observed over a wide temperature range above approximately 10 mA. Such temperature-independent resistance is a characteristic feature of zero-gap semimetals in organic conductors [17, 18]. Fig. S6c is an Arrhenius plot of Fig.S6a, and the calculated gap size Δ for each excitation current is plotted in Fig. S6d. Indeed, Δ decreases with increasing current value and asymptotically approaches zero. Consequently, the current-induced metallic state is a zero-gap Dirac semimetal.

As discussed in the main text, negative MR was observed in the current-induced state, which indicates the bulk Dirac semimetal state. To confirm that the negative MR is intrinsic and not due to extrinsic origin such as heat generation by eddy currents in pulsed magnetic fields or the other problems in the measurement setup, we performed MR measurement with the in-plane configuration in a superconducting magnet. Fig. S7 shows the results of the MR measurement using two values of excitation current, 0.001 mA and 8 mA. A positive MR is observed using the small excitation current, while a negative MR is observed using the large excitation current. This result demonstrates that the negative MR in the current-induced metallic phase is reproducible and not due to extrinsic origins. For the data taken with 0.001 mA, a small negative MR was observed below 1 T, which can be attributed to the weak localization effect.

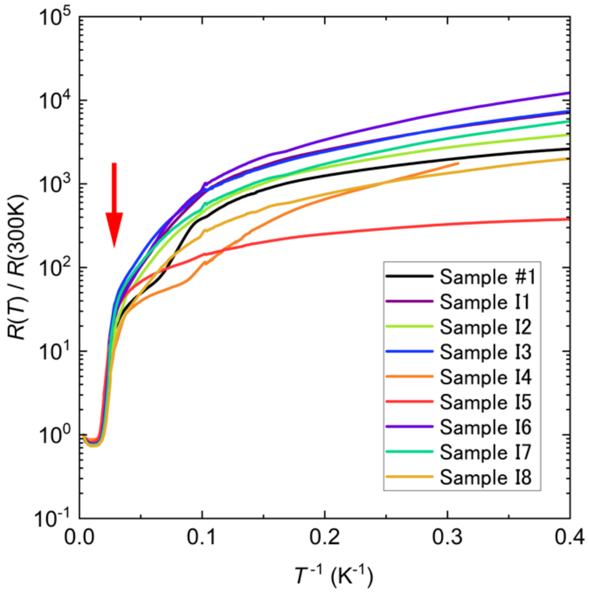

Figure S1 | Arrhenius plot of the in-plane resistance on α-(BETS)$_2$I$_3$ for Sample #1 and Sample I1-I8. The resistance is normalized by the value at room temperature. The red arrow corresponds to the crossover temperature T = 35 K. Below 35 K, the sample dependence of resistance can be clearly observed, indicating the surface conduction in low temperatures.

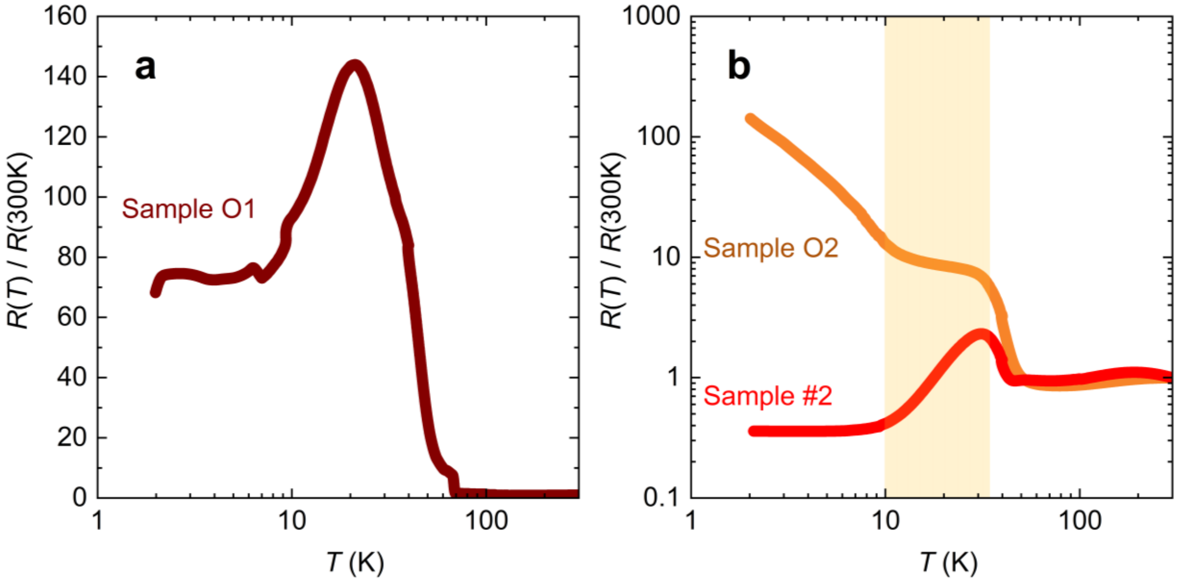

Figure S2 | Sample dependence of the out of plane resistance on α-$(BETS)_2I_3$. a Example of the sample showing the resistance drop at approximately 30 K (Sample O1). **b** Example of the sample not showing the resistance drop at approximately 35 K (Sample O2). The temperature dependence of Sample O2 is similar to that of Sample #2 above 35 K, where the contribution of the surface conduction is negligibly small. Below 35 K, where the surface conduction emerges, the temperature dependence of resistance is quite different between Sample O2 and Sample #2.

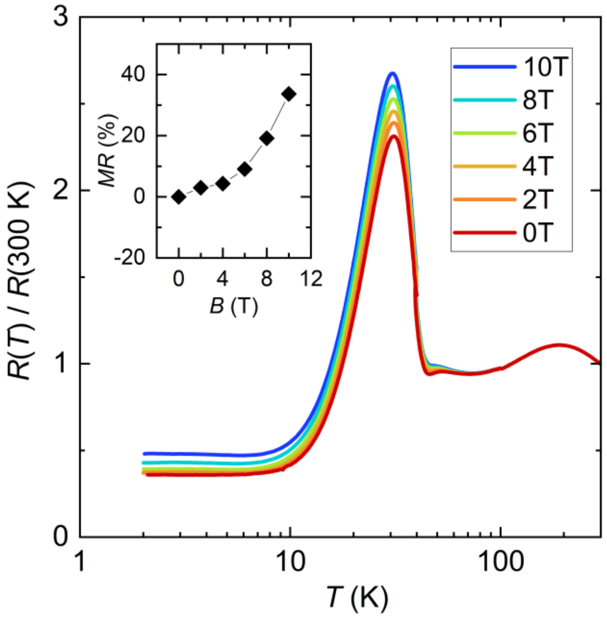

Figure S3 | MRs in *I* // *B* configuration. Temperature dependence of the out-of-plane resistance (Sample #2) under magnetic fields of 0, 2, 4, 6, 8, and 10 T. The magnetic field was applied perpendicular to the *ab*-plane (*B* // *c*-axis). The resistance monotonically increases with increasing fields. A negative MR cannot be observed in the out-of-plane resistance measurements. Inset: Magnitude of the positive MR at 2 K. The MR increases quadratically with increasing magnetic fields.

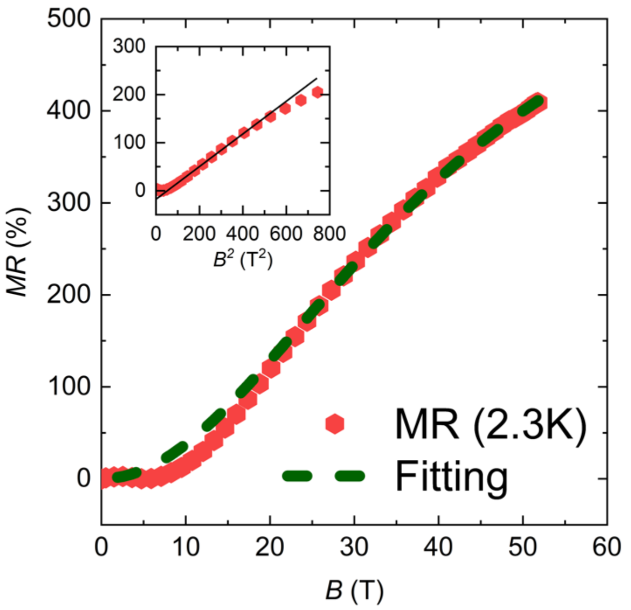

Figure S4 | Positive MR at 2.3 K under the $I \perp B$ configuration in Sample #1. The green dashed line is the fitting curve using equation (1). The parameters used in the fit are $r_0 = 1.31 \times 10^{-3}$, $R_h = 5.05 \times 10^{-5}$, and $R_e = 1.14 \times 10^{-4}$. Inset: MR vs B^2 plot. In the weak magnetic field region below 20 T, MR follows B^2 (the black solid line).

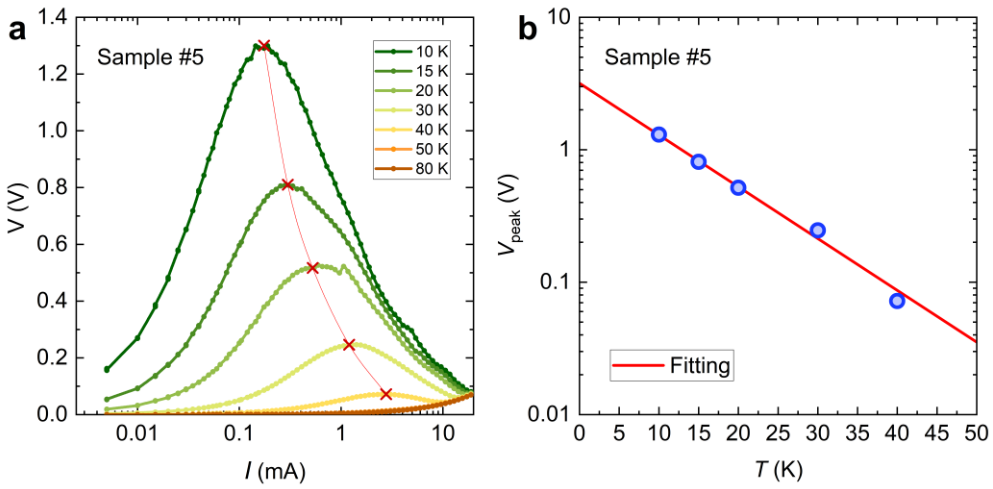

Figure S5 | Current-voltage character of α-$(BETS)_2I_3$. a Enlarged figure of the current-voltage characteristic of Sample #4. The red dashed line points out V_{peak}. **b** V_{peak} versus T. The red solid line is the fitting curve $V_{peak}(T) = V_0\exp(-T/T_0)$ using $V_0 = 3.18$ V and $T_0 = 11.1$ K.

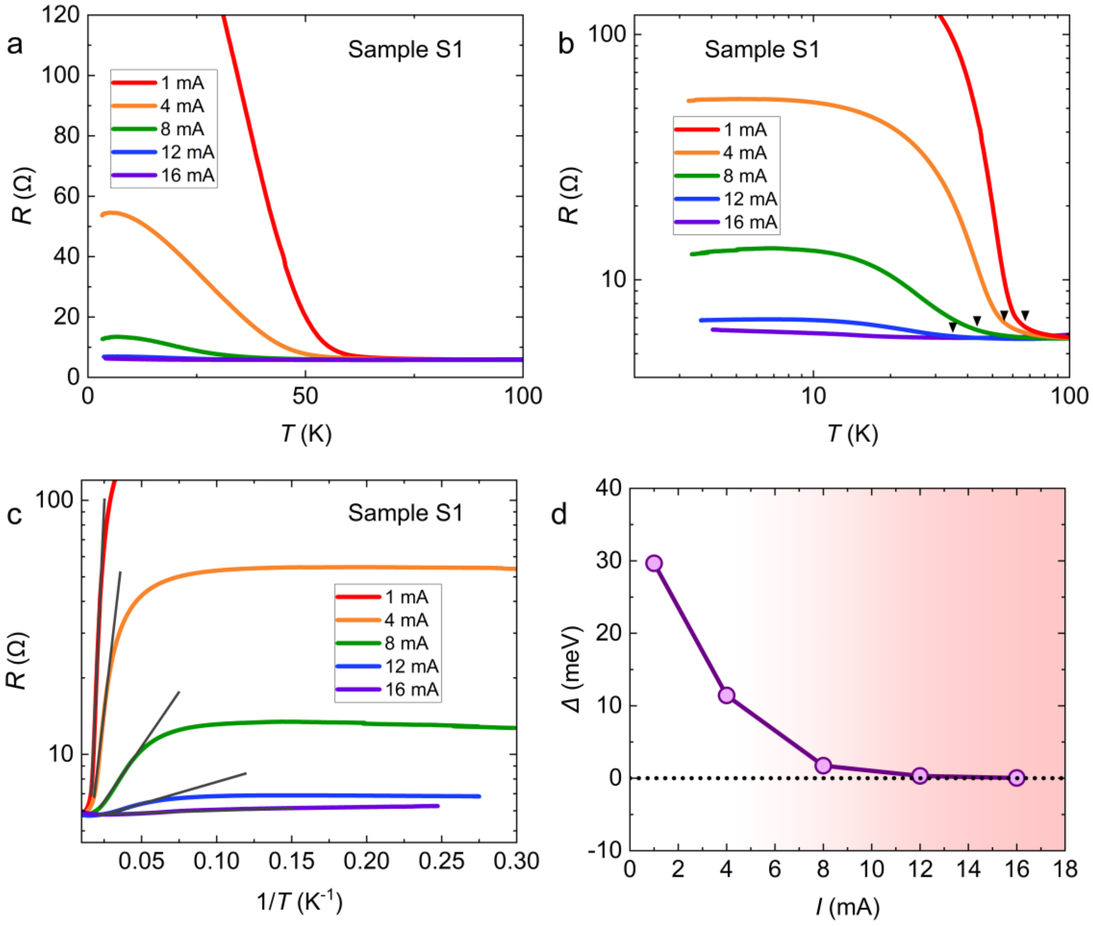

Figure S6 | Temperature dependence of the in-plane resistance of α-$(BETS)_2I_3$ (Sample S1) measured by using a variety of excitation currents. a The temperature-independent resistance is observed in the excitation currents of 12 and 16 mA, which is a typical feature of zero-gap systems. Inset: current dependence of the gap size estimated by each resistance curve. **b** The log-log plot of **a**. The resistance curve obtained with the excitation current of 16 mA is almost temperature-independent between 100 and 2 K. The black triangles indicate the onset temperature of the resistance increase. The onset temperature seems to decrease with increasing excitation current. **c** The Arrhenius plot of **a**. The black solid lines represent linear fittings of the resistance curves. **d** The current dependence of the gap size estimated by the Arrhenius plots.

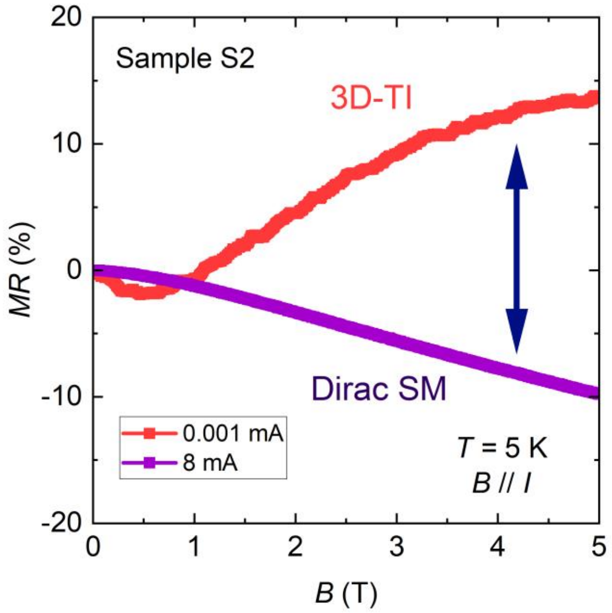

Figure S7 | Current switching of the MR in α-$(BETS)_2I_3$ (Sample S2) for the in-plane resistance measured in steady magnetic fields.

Table S1 | Dimensions (length, width, and thickness) of the measured samples #1~#6. Sample #3 and #4, for which in-plane resistance measurements by the inverted and non-local configurations were performed, are not listed here because its length and cross-sectional area are difficult to be defined. The thickness of Sample #3 and Sample #4 were 30 and 10 μm, respectively.

Sample name	Length (μm)	Width (μm)	Thickness (μm)
#1	2.0×10^{2}	1.8×10^{3}	5.0×10^{1}
#2	1.0×10^{3}	5.6×10^{2}	1.0×10^{1}
#5	1.6×10^{2}	7.2×10^{2}	3.0×10^{1}
#6	1.4×10^{2}	7.2×10^{2}	1.0×10^{1}
#7	1.0×10^{2}	9.0×10^{2}	4.0×10^{0}

Supplementary References

1. Phelan, W. A. et al. Correlation between Bulk Thermodynamic Measurements and the Low-Temperature-Resistance Plateau in SmB_6. *Phys. Rev. X* **4**, 031012 (2014). 10.1103/PhysRevX.4.031012.

2. Taskin, A. A., Ren, Z., Sasaki, S., Segawa, K. & Ando, Y. Observation of Dirac holes and electrons in a topological insulator. *Phys. Rev. Lett.* **107**, 016801 (2011). 10.1103/PhysRevLett.107.016801, Pubmed:21797561.

3. Zhang, X. et al. Hybridization, inter-ion correlation, and surface states in the Kondo insulator SmB_6. *Phys. Rev. X* **3**, 011011 (2013).

4. Wolgast, S. et al. Low-temperature surface conduction in the Kondo insulator SmB_6. *Phys. Rev. B* **88**, 180405(R) (2013). 10.1103/PhysRevB.88.180405.

5. Kim, D. J. et al. Surface Hall effect and nonlocal transport in SmB_6: evidence for surface conduction. *Sci. Rep.* **3**, 3150 (2013). 10.1038/srep03150, Pubmed:24193196.

6. Liang, T. et al. Ultrahigh mobility and giant magnetoresistance in the Dirac semimetal Cd_3As_2. *Nat. Mater.* **14**, 280-284 (2015). 10.1038/nmat4143.

7. Shekhar, C. et al. Extremely large magnetoresistance and ultrahigh mobility in the topological Weyl semimetal candidate NbP. *Nat. Phys.* **11**, 645-649 (2015). 10.1038/nphys3372.

8. Tajima, N., Sugawara, S., Kato, R., Nishio, Y. & Kajita, K. Effect of the zero-mode landau level on interlayer magnetoresistance in multilayer massless Dirac fermion systems. *Phys. Rev. Lett.* **102**, 176403 (2009). 10.1103/PhysRevLett.102.176403.

9. Du, X., Tsai, S. W., Maslov, D. L. & Hebard, A. F. Metal-insulator-like behavior in semimetallic bismuth and graphite. *Phys. Rev. Lett.* **94**, 166601 (2005). 10.1103/PhysRevLett.94.166601, Pubmed:15904256.

10. Fauqué, B., Vignolle, B., Proust, C., Issi, J.-P. & Behnia, K. Electronic instability in bismuth far beyond the quantum limit. *New J. Phys.* **11**, 113012 (2009). 10.1088/1367-2630/11/11/113012.

11. Kopelevich, Y. et al. Reentrant metallic behavior of graphite in the quantum limit. *Phys. Rev. Lett.* **90**, 156402 (2003). 10.1103/PhysRevLett.90.156402, Pubmed:12732058.

12. Huynh, K. K., Tanabe, Y. & Tanigaki, K. Both electron and hole Dirac cone states in $Ba(FeAs)_2$ confirmed by magnetoresistance. *Phys. Rev. Lett.* **106**, 217004 (2011). 10.1103/PhysRevLett.106.217004, Pubmed:21699332.

13. Ali, M. N. et al. Large, non-saturating magnetoresistance in WTe_2. *Nature* **514**, 205–208 (2014). 10.1038/nature13763, Pubmed:25219849.

14. Nakano, M., et al. "Collective bulk carrier delocalization driven by electrostatic surface charge accumulation." *Nature* **487**(7408) 459-462 (2012).

15. Shukla, N. et al. "Electrically induced insulator to metal transition in epitaxial $SmNiO_3$ thin films." *Appl. Phys. Lett.* **105**(1), 012108 (2014).

16. Ajisaka, S. et al. "Nonequilibrium peierls transition." *Prog. Theor. Phys.* **121**(6), 1289-1319 (2009).

17. Tajima, N. & Kajita, K. Experimental study of organic zero-gap conductor α-$(BEDT\text{-}TTF)_2I_3$. *Sci. Technol. Adv. Mater.* **10**, 024308 (2009). 10.1088/1468-6996/10/2/024308, Pubmed:27877281.

18. Kato, R., Cui, H., Tsumuraya, T., Miyazaki, T. & Suzumura, Y. Emergence of the Dirac Electron System in a Single-Component Molecular Conductor under High Pressure. *J. Am. Chem. Soc.* **139**, 1770–1773 (2017). 10.1021/jacs.6b12187, Pubmed:28121146.
